# Photodynamic Therapy Using Hippo Pathway Inhibitor Verteporfin: A Potential Dual Mechanistic Approach in Treatment of Soft Tissue Sarcomas

**DOI:** 10.3390/cancers13040675

**Published:** 2021-02-08

**Authors:** Jeffrey D. Rytlewski, Nicholas Scalora, Keith Garcia, Munir Tanas, Fatima Toor, Benjamin Miller, Bryan Allen, Mohammed Milhem, Varun Monga

**Affiliations:** 1 Department of Internal Medicine, University of Iowa, Iowa City, IA 52242, USA; jeffrey-rytlewski@uiowa.edu; 2 Department of Pathology, University of Iowa, Iowa City, IA 52242, USA; nicholas-scalora@uiowa.edu (N.S.); keith-garcia@uiowa.edu (K.G.); munir-tanas@uiowa.edu (M.T.); 3 Department of Electrical and Computer Engineering, University of Iowa Technology Institute, University of Iowa, Iowa City, IA 52242, USA; fatima-toor@uiowa.edu; 4 Department of Orthopedic Surgery, University of Iowa, Iowa City, IA 52242, USA; benjamin-j-miller@uiowa.edu; 5 Department of Radiation Oncology, University of Iowa, Iowa City, IA 52242, USA; bryan-allen@uiowa.edu; 6 Division of Hematology, Oncology, and Blood & Marrow Transplant, Department of Internal Medicine, University of Iowa, Iowa City, IA 52242, USA; mohammed-milhem@uiowa.edu

**Keywords:** Hippo pathway, soft tissue sarcoma, verteporfin, photodynamic therapy, YAP/TAZ

## Abstract

**Simple Summary:**

Advanced sarcomas have yet to undergo improved outcomes seen in other cancer subtypes. Verteporfin has the potential to show landmark change in sarcoma due to its anti-proliferative properties: inhibition of the Hippo pathway and as photodynamic therapy. The effect of verteporfin on the Hippo pathway is reviewed specifically in the setting of sarcoma due to increased activation of this pathway in multiple subtypes. Role and efficacy of photodynamic therapy in other malignancies is also reviewed, with additional discussion of preclinical studies demonstrating synergistic effects of photodynamic therapy within current sarcoma standard of care treatment. Future investigations of the feasibility of incorporating verteporfin into sarcoma treatment are discussed.

**Abstract:**

Sarcoma is a widely varied and devastating oncological subtype, with overall five-year survival of 65% that drops to 16% with the presence of metastatic disease at diagnosis. Standard of care for localized sarcomas is predicated on local control with wide-local resection and radiation therapy, or, less commonly, chemotherapy, depending on tumor subtype. Verteporfin has the potential to be incorporated into this standard of care due to its unique molecular properties: inhibition of the upregulated Hippo pathway that frequently drives soft tissue sarcoma and photodynamic therapy-mediated necrosis due to oxidative damage. The initial anti-proliferative effect of verteporfin is mediated via binding and dissociation of YAP/TEAD proteins from the nucleus, ultimately leading to decreased cell proliferation as demonstrated in multiple in vitro studies. This effect has the potential to be compounded with use of photodynamic therapy to directly induce cellular necrosis with use of a clinical laser. Photodynamic therapy has been incorporated into multiple malignancies and has the potential to be incorporated into sarcoma treatment.

## 1. Introduction

Sometimes unexpectedly, repurposing forgotten or outdated knowledge reveals progress for exciting breakthroughs. One example in the field of oncology is photodynamic therapy (PDT), a technology utilized as remotely as ancient Egypt and ancient Greece [1]. PDT uses light to activate a previously biologically inert photosensitizer [2,3]. Structural factors of the photosensitizer cause increased absorption of a specific wavelength of light, electron emission, and multiple established mechanisms of tumor destruction [2,3,4]. One such mechanism is the formation of reactive oxygen species (ROS) that lead to overwhelming oxidative stress and apoptosis. Wang et al. demonstrated that tumor cell’s ability to reduce oxidative stress was inversely correlated to effectiveness of PDT [2,4,5]. This mechanism was additionally supported by the fact that the cytotoxic effect of PDT was eliminated by inducing tissue hypoxia via clamping of the blood supply [6]. When photosensitizer has accumulated in the tumor vasculature, as opposed to tumor body, activation leads to destruction of associated vasculature and infarction of the tumor bed [2,4]. A third supported mechanism includes the release of antigens and damage-associated molecular patterns (DAMPs) during tumor necrosis, which potentiates an immune response against tumor antigens [2,7]. The extent of cytotoxicity due to the above mechanisms is dependent on the ratio of Type I to Type II ROS reactions, location of the photosensitizer within the tissue, total dose of photosensitizer administered, total light exposure dose, total light fluence rate, oxygen availability within the tissue, and time of administration between photosensitizer and light exposure [2]. Because PDT is not mechanistically selective, multiple studies have demonstrated that normal tissue that has been exposed to PDT is able to undergo healing without significant structure or function loss [8,9].

## 2. Photodynamic Therapy in Clinical Studies

As PDT demonstrated promise as well as increased feasibility with the advent of laser technology, there have been a variety of studies undertaken to determine the effectiveness and safety of using PDT in treating cancers. Phase I trials in the setting of localized prostate adenocarcinoma (WST11) using PDT found minimal side effects with respect to urinary retention and sexual function, and ultimately moved into phase II trials [10,11]. An early phase trial using hexaminolevulinate photosensitizer in bladder cancer was found to have serious adverse effects of hematuria and irritative bladder/urgency syndrome related to PDT but was ultimately concluded to be tolerable after dose adjustment [12]. Twenty-nine patients were enrolled in a phase I trial dose escalation study using a boronated photosensitizer following surgical resection of glioma [13]. The only severe adverse effect reported was thrombocytopenia at the increased photosensitizer dose in two patients; both recovered. Thrombocytopenia was considered a dose-limiting toxicity in this instance; however, the agent was considered tolerable. Phase I trials performed in laryngeal, esophageal, nasopharyngeal, and endobronchial carcinomas demonstrated parallel results: dose-limited severe adverse effects that warranted further investigation in phase II trials [14,15,16,17,18].

The multifactorial nature of PDT therapy delivery necessitates highly specified protocols to maintain the efficacy seen in preclinical settings. Huggett et al. performed a phase I/II trial evaluating the efficacy of the photosensitizer verteporfin in pancreatic adenocarcinoma that underscored the importance of accurate delivery of light exposure to exclusively identified tumor tissue [19]. The trial enrolled 15 patients with tumors deemed unresectable at diagnosis for neoadjuvant PDT and chemotherapy. The primary endpoint of the trial was safety, with an additional endpoint being documentation of tumor necrosis. The degree of necrosis increased with increasing administration of light intensity, with one patient becoming a candidate for a Whipple procedure. Post-study analysis demonstrated technical difficulties with the light fibers, with emission up to 1 cm proximal to the fiberoptic tip causing delivery of therapy to unwanted areas. Most patients reported abdominal pain following PDT without lipase elevation.

Hahn et al. evaluated the PDT agent photofrin delivered during surgical resection of primary tumors in metastatic ovarian, gastrointestinal, and peritoneal sarcoma [20]. This phase II trial enrolling 100 patients looked at endpoints of complete response, failure-free survival, and overall survival. Only 12% of patients achieved complete response as assessed by laparoscopy at three months, with 100% showing progressive disease with a median follow-up of 46 months. Major toxicities were related to fluid shifts attributed to capillary leak syndrome requiring massive fluid resuscitation (14%), ARDS (4%), and postoperative ileus (2%); two patients died in the postoperative period from myocardial infarction and sepsis. While PDT techniques were considered feasible in this patient cohort, they did not correlate with improved response and were associated with significant complications. After post-study analysis of tissue concentrations of the photosensitizer, the authors postulated that lack of response could be attributed to heterogeneity and lack of specificity in photosensitizer uptake as well as tissue variance in light absorption.

Multiple phase II clinical trials have evaluated the efficacy of PDT in the setting of cholangiocarcinoma, demonstrating promise in the mechanistic potential of PDT as well as its potential for synergistic effects with current oncological treatment regimens [21,22]. Including case reports, the potential benefits of PDT in the setting of cholangiocarcinoma were demonstrated in the 1990s. Survival benefits were demonstrated in several studies [22,23,24]. These were the first of a series of phase II trials that demonstrated the efficacy of endoscopically delivered PDT in the setting of unresectable cholangiocarcinoma, leading to a best practice recommendation in 2004 to offer PDT to appropriate patients following proper staging and surgical evaluation [22,25]. Further developments in the field of cholangiocarcinoma-specific PDT include a phase II trial evaluating an improved photosensitizer, as well as a phase II clinical trial demonstrating a synergistic effect of chemotherapy and PDT leading to survival benefit over PDT alone [21,26]. The advances seen in the field of cholangiocarcinoma lend credence to the idea that, once mechanistic underpinnings are identified, PDT may become an effective tool in the oncologist’s arsenal in the near future.

## 3. Considerations in Photodynamic Therapy Delivery in Soft Tissue Sarcomas

One of the key challenges of PDT in clinical use is the controlled delivery of light in order to maximize therapeutic benefit while minimizing side effects. As seen in experimentation performed by Huggett et al., more light delivered led to a higher level of necrosis, but the extent of cellular damage did not correlate to the dose of light given and was unexpectedly high [19]. These data underscored the difficulty in predicting treatment response, despite knowing several variables related to PDT delivery. Furthermore, Huggett and colleagues reported that in patients treated with multiple rounds of light therapy, there was unexpected inflammation, which was later attributed to inappropriate light emission from the fibers [19].

In a report by Bown et al., solid organ was dosed unintentionally in a patient when a fiber moved after positioning [8]. Despite receiving inadvertent treatment, the area eventually healed and recovered. These observations may indicate that PDT is not highly tumor selective, but that the areas of unintended treatment may not experience long term damage or loss of function [8,9]. With regards to healthy muscle and bone, likely to undergo unintended PDT treatment if soft tissue sarcoma is treated, studies in the dental and orthopedic fields have shown low dose PDT to be a stimulus for growth and repair, reiterating the possibility for healthy tissues to repair themselves [27,28].

Adverse events (AEs) have been reported in using verteporfin as a PDT in cancer treatments. In a single arm single site Phase 1 study in 16 patients with recurrent resectable head and neck cancer, grade 1 and 2 edema lasting seven to 10 days and pain lasting two to four weeks was reported. These AEs were possibly attributed to PDT. There were phototoxicity reactions including grade 2 photophobia, which was likely related to prolonged exposure to operating room spotlights and two skin burns reported due to prolonged exposure from pulse oximeter for an extended period [29]. A number of other short term side effects such as local site skin reactions such as erythema, edema, desquamation, or pustules, urticaria, contact dermatitis, and long term side effects such as pigmentary changes and potentially secondary skin cancers have been reported when PDT is used for treatment of non-melanomatous skin cancers [30].

## 4. Why Use Photodynamic Therapy in Soft Tissue Sarcoma?

**Figure 1:**
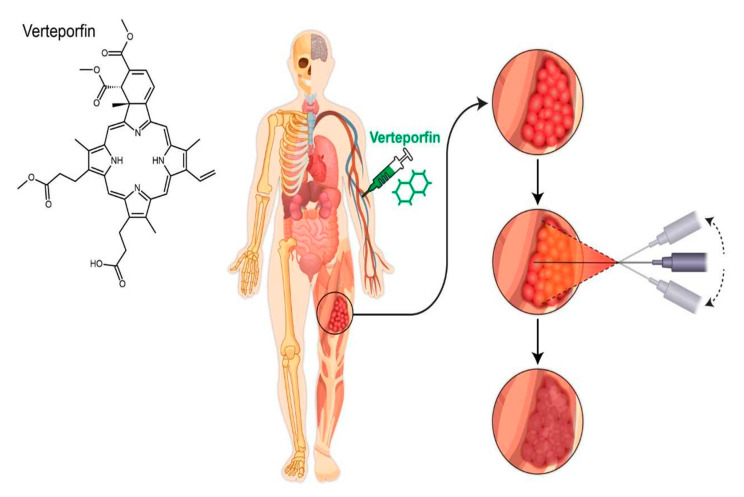
A theoretical demonstration of photodynamic therapy in sarcoma with intravenous verteporfin followed by transcutaneous laser light delivery leading to tumor necrosis.

One field in which similar advancement would be welcome is soft tissue sarcoma (STS), in which the current standard treatment of localized disease still results in relapse rates of 20–30% within three years [31]. According to the SEER database, five-year overall survival rates of soft tissue sarcomas are 65%, with the presence of distant disease at diagnosis decreasing survival to 16% [32]. Current management plans for localized STS utilize combination neoadjuvant radiation, with the possible addition of chemotherapy in certain sarcoma subtypes, prior to wide local excision surgical resection, with outcomes improved at designated treatment centers [31,33]. The potential addition of an effective therapeutic tool such as photodynamic therapy (Figure 1) could add substantial benefit for patients in achieving higher pathological response and to achieve surgically negative margins. A review performed by Zhang et al. examined multiple pre-clinical and clinical studies evaluating the use of PDT in the setting of osteosarcomas, with the majority of studies referenced utilizing acridine-orange [34]. One study referenced by Kusuzaki et al. specifically looked at using PDT with acridine orange as a surgical surrogate to achieve surgical margins, finding that recurrence rate was less than 10% and functionality was maintained [34,35]. Final conclusions by Zhang et al. felt that once the issue of light delivery was circumvented, PDT offered promise in management of sarcomas [34].

Given that PDT requires the use of a molecular potentiator to begin its cytotoxic effects, it allows for additional opportunity to evolve and be used in different clinical settings involving STS management. For example, PDT with verteporfin can be utilized intraoperatively to improve negative margin rate in STS located around critical structures such as sciatic nerve or femoral artery during surgical resection. Although there is a potential for damage to surrounding normal tissue, this can be mitigated with careful planning the wavelength of light used with known depth of penetration.

Neoadjuvant radiation is routinely used for management of large, high risk soft tissue extremity sarcomas [33]. Higher rate of hyalinization and fibrosis is associated with better overall outcomes in STS [36]. PDT in combination with radiation has shown to enhance radiation sensitivity in radiation induced fibrosarcoma-1 tumors and delayed tumor regrowth in pre-clinical studies [37]. Hence this combination of PDT with standard external beam radiation can be used in the management of STS to improve clinical outcomes.

## 5. Hippo Pathway in Sarcoma and Role of Verteporfin

The Hippo pathway was stumbled upon during investigations related to organogenesis, and the intrinsic regulatory mechanisms affecting end-organ size [38]. Originally discovered in *Drosophila*, the Hippo pathway consists of a series of kinase cascades with impacts in tissue regeneration and malignancy through its downstream transcriptional activation via Yorkie (Yki) working in concert with Scalloped (Sd) proteins [38]. In mammalian cells, YAP and TAZ are the orthologues of Yki and are transcriptional coactivators and oncoproteins that lack DNA binding domains of their own. To drive transcription, they must bind to other transcription factors containing DNA binding domains such as the TEAD transcription factors, which are orthologues of Sd [38].

The Hippo pathway is activated under multiple different conditions, some of which include cells in a detached state or contact inhibition from neighboring cells. Once activated, the serine/threonine kinases MST1/2 phosphorylate LATS1/2, which ultimately phosphorylate TAZ and YAP, leading to their degradation [39]. In Drosophila development, it has been shown that Hippo activation leads to phosphorylation of DIAP1, an inhibitor of apoptosis, thereby promoting apoptosis. While the underlying mechanisms in which TAZ and YAP lead to apoptosis in mammalian cells are not well understood, a constitutively active form of YAP results in resistance to anoikis, a form of programmed cell death, upon detachment [40]. This suggests that when appropriately activated, the upstream Hippo pathway kinases demonstrate tumor suppressor effects, whereas dysregulation within the pathway contributes to oncogenic potential by activation of TAZ and YAP [41].

In addition to their tumor promoting capabilities, the Hippo pathway end effectors TAZ and YAP have also been shown to have the capacity to drive metastasis in a broad spectrum of cancers such as breast, melanoma, lung, gastric, and colorectal, as well as sarcomas [42]. This is especially true in STS, where immunohistochemical analysis has shown that YAP and TAZ are commonly activated (50% and 66%, respectively) across different histological types of sarcoma and correlate with increased tumor grade and decreased overall survival [43]. Isfort et al. expanded upon this work by observing 486 sarcomas and reported TAZ activation in 33% and YAP activation in 53% [44]. In vitro studies show that targeting the YAP/TEAD complex with verteporfin reduces anchorage independent growth in soft agar [43] treatment with verteporfin in two different in vivo models of hepatomegaly, either induced by YAP overexpression or NF2 inactivation (negative regulator of YAP), showed considerable reduction in liver overgrowth [45]. These data show promise for the use of verteporfin in oncological management.

Furthermore, TAZ and YAP have been shown to drive tumorigenesis in many sarcomas in pre-clinical studies. A PAX3-FOXO1A fusion protein increases TAZ expression in an alveolar rhabdomyosarcoma, and xenograft mouse models show TAZ suppression inhibits tumor growth via a decrease in proliferation and a slight increase in apoptosis [46]. In mice, hyperactivated YAP expressed in activated satellite cells drives formation of embryonal rhabdomyosarcoma in a TEAD1 dependent manner [47]. Other studies have shown that the YAP complexes with TEAD1 and is an oncogenic driver in undifferentiated pleomorphic sarcoma [48]. SS18-SSX fusion protein has been established as the oncogenic driver of synovial sarcomas. Isfort et al. described a molecular based mechanism of YAP/TAZ activation in synovial sarcoma that involves IGF-II/IGF-1R signaling loop, which leads to aberrant LATS1 and MOB1 [49]. Verteporfin treatment lead to modulation of YAP/TAZ-TEAD mediated transcriptional activity, resulting in significant growth inhibition both in vitro and in vivo [49]. In myxoid liposarcoma (MLS), another subtype of soft tissue sarcoma known to be driven by FUS-DDITs fusion gene has been shown to promote YAP1 expression. When YAP1 activity was inhibited using verteporfin the growth of MLS cells both in vitro and in vivo was impaired, thereby making overactive YAP1 as a potential therapeutic target in MLS [50]. Taken together verteporfin with its non-photodynamic effect can potentially be used in management of sarcomas.

**Figure 2:**
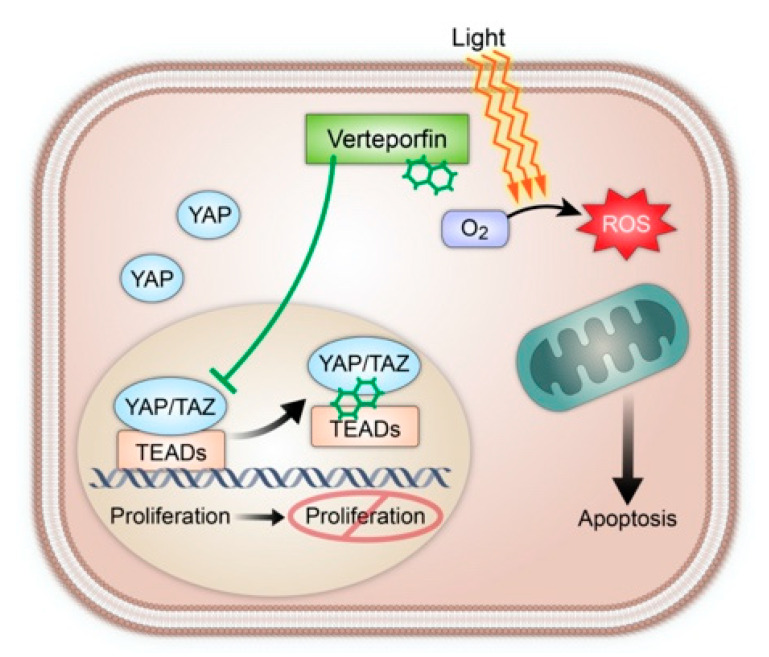
A demonstration of Verteporfin dual mechanism via YAP/TAZ interaction leading to decreased cellular proliferation (left) and induction of cellular apoptosis following light activation leading to oxidative damage (right).

The primary cause of mortality in sarcoma patients is metastasis, resulting in a five-year survival rate of approximately 15%, making it an essential target for therapeutic intervention [51]. TAZ/YAP-TEAD interaction, as seen in multiple STS subtypes, represents an exciting therapeutic target for localized and potentially metastatic sarcomas. Expanded upon by Wei et al. in their recent review article, verteporfin has the potential to contribute as a chemosensitizer and inhibit autophagy in order to decrease chemotherapy resistance, and has demonstrated YAP independent cytotoxic effects in vitro [52]. Hence, this effect on YAP/TAZ inhibition together with standard chemotherapy drugs approved in sarcoma management such as anthracyclines leads to easy selection of verteporfin for future clinical studies (Figure 2).

## 6. Proposed Delivery of Light Source in Soft Tissue Sarcoma

PDT requires the presence of three components: light, photosensitizer, and oxygen. A wide range of light sources can be used for PDT, including light emitting diodes, lasers, and fluorescent lamps [53]. The light source is typically chosen based on photosensitizer absorption, disease characteristics, and costs [53,54]. Red (wavelength = 600–750 nm) and infrared light (wavelength = 750–800 nm) best penetrate tissues, up to 5 mm in depth, but that may not be sufficient for large volumetric cancers, such as sarcoma.

Furthermore, recently there have been innovative approaches reported to address the low penetration of light during PDT. Bansal et al. reported a wireless photonic approach to PDT using a miniaturized (30 mg, 15 mm^3^) implantable device and wireless powering system for light delivery [55]. The team demonstrated the therapeutic efficacy of their approach by activating the photosensitizer (chlorin e6) through thick (>3 cm) tissues inaccessible by direct illumination, and by delivering multiple controlled doses of light to suppress tumor growth in vivo in animal cancer models. Kim et al. reported an ultrasonically powered implantable microlight source, μLight, which enables in-situ localized light delivery to deep-seated solid tumors [56]. In vivo tests in mice implanted with 4T1-induced tumors (breast cancer) showed light delivery capability at therapeutic dose levels. Overall, results indicated that implanting multiple µLights and operating them for 1–2 h can achieve cytotoxicity levels comparable to clinically reported cases using external light sources. These implantable devices could be one approach to treating sarcomas through PDT.

## 7. Conclusions and Future Directions

As seen in prior clinical trials, especially once appropriate protocols and treatment deliveries are identified, PDT with verteporfin offers tremendous opportunity for progression in the field of oncology. Treatment delivery scaling does present logistical challenges and tends to favor minimally invasive malignancies with simpler procedural access (e.g., transurethral, bronchoscopy, endoscopy).

**Figure 3:**
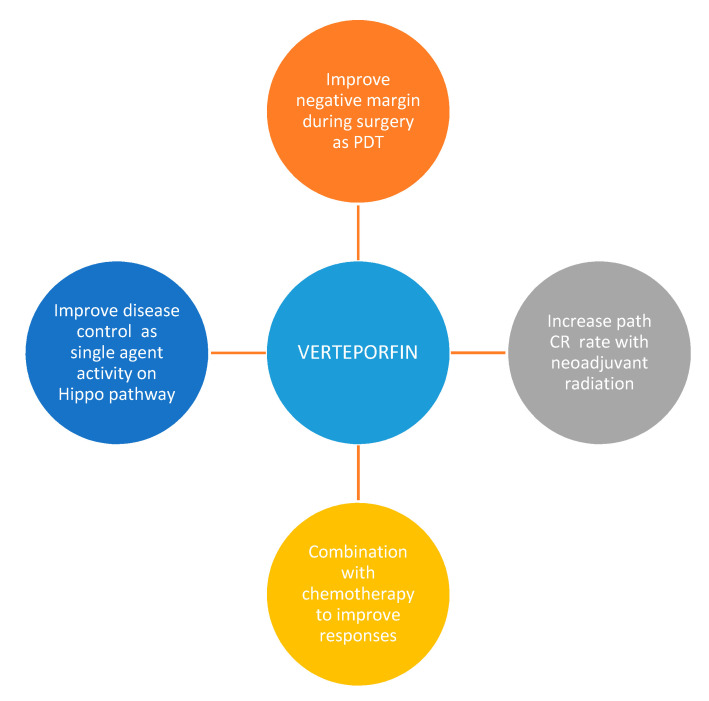
Verteporfin as PDT and as a Hippo pathway inhibitor can be used clinically in the management of localize and advanced STS. Path CR: Pathological complete response. PDT: Photodynamic therapy.

One of the more encouraging aspects of verteporfin therapy is its possible synergistic effects with current standard of care treatments in STS, with doxorubicin and radiation therapy. This coupled with the anti-proliferative effect shown in several in vivo studies even in the absence of PDT would by itself be encouraging to explore in advanced sarcomas given the degree of limited treatment options and the morbidity and mortality experienced with this cancer. However, given the mechanistic promise verteporfin offers as well as the wealth of preclinical studies demonstrating its worth as a translational agent, exploring minimally invasive light therapy delivery mechanisms in concert with pharmaceutical administration is an appropriate step forward (Figure 3).

We propose to initially assess single agent verteporfin in a dose finding Phase 1b/2 study to determine its safety in relapsed refractory metastatic sarcomas (both STS and bone sarcomas). Verteporfin efficacy will be determined using RECIST criteria in metastatic target lesions and will be correlated with YAP/TAZ expression on the archival tumor specimen. If tumor response correlates with the YAP/TAZ expression, this may be considered as an eligibility criterion in a future larger randomized trial. Ideally, the effect of verteporfin on YAP/TAZ dependent gene expression should be tested on metastatic tumors; however, as many STS tumors metastasize to the lungs, tumor biopsies can be challenging. As an alternative approach, YAP/TAZ gene expression could be evaluated in peripheral blood mononuclear cells obtained prior to and serially during verteporfin treatment as a surrogate marker. Once the single agent activity and toxicity is determined, then combination strategies with chemotherapy or targeted therapy or immunotherapy could be explored. Verteporfin as a PDT agent can be explored in the preoperative setting together with standard of care external beam radiation therapy to the primary tumor located in either the extremity or trunk. However, one of the biggest challenges is the effective light delivery at varying depths of the tumor tissue using optical fibers. Recently, interstitial PDT has been developed to address this challenge by using multiple optical fibers during PDT, which are precisely inserted using dosimetry, at different locations of the large tumor volume [57]. Pre-clinical sarcoma models will help determine the effectiveness of this strategy. Another potential clinical trial setting could be intraoperative, during which verteporfin can be infused at the end of surgery and appropriate wavelength of light can be exposed to the surgical bed and surgical margins with the intent to obtain negative surgical margins.

Considerations of light therapy in sarcoma include the depth to which the light exposure must reach, the assurance the entire tumor is treated, and minimizing possible harm to the surrounding normal tissue induced by this intervention. Discovering novel light delivery techniques with fiberoptic light sources to go along with verteporfin use would be essential, as transcutaneous delivery methods in other cancer trials cannot be reproduced to the tissue depth needed for similar success in deep seated sarcomas. Despite these challenges, the next steps forward will likely prove beneficial for both provider and patient.

## Data Availability

No new data were created or analyzed in this study. Data sharing is not applicable to this article.
